# The State of Cellular Adoptive Immunotherapy for Neuroblastoma and Other Pediatric Solid Tumors

**DOI:** 10.3389/fimmu.2017.01640

**Published:** 2017-11-24

**Authors:** Thanh-Phuong Le, To-Ha Thai

**Affiliations:** ^1^Department of Pathology, Beth Israel Deaconess Medical Center, Harvard Medical School, Boston, MA, United States

**Keywords:** pediatric solid tumors, recurrent/refractory/relapsed neuroblastoma, adoptive T-cell therapy, immune cell-based therapy, natural killer cells, *Cbx3*/HP1γ, CD4^+^ regulatory T cells, effector CD8^+^ T cells

## Abstract

Research on adult cancer immunotherapy is proceeding at a rapid pace resulting in an impressive success rate exemplified by a few high profile cases. However, this momentum is not readily extended to pediatric immunotherapy, and it is not for lack of trying. Though reasons for the slower advance are not apparent, some issues can be raised. Pediatric cancer patients represent a distinct demographic group whose immune system is inherently different from that of mature adults. Treating pediatric patients with immunotherapy designed for adults may not yield objective clinical responses. Here, we will present an update on adoptive T-cell and natural killer-cell therapies for neuroblastoma and other childhood solid tumors. Additionally, we will delineate key differences between human fetal/neonatal and adult immune systems. We hope this will generate interests leading to the discussion of potential future directions for improving adoptive cancer immunotherapy for children.

## Introduction

In the past few decades, pivotal studies have yielded invaluable information on pediatric oncology leading to the formulation of standard therapies still being performed today (1–9). However, it is now evident that the majority of resistant, metastatic, recurrent/refractory tumors are non-responsive to conventional therapies (10–20). In addition, current approaches often rely on non-specific, cytotoxic chemotherapy and/or radiotherapy that result in long lasting, debilitating toxicities, and in some instance morbidity (21–26). Therefore, there is a need to explore new avenues to eradicate pediatric cancers.

Two seminal reports, published by the surgeon and cancer researcher William Bradley Coley in the late 19th century, show sarcoma tumor regression in patients repeatedly immunized with live or killed streptococcus bacteria (27, 28). His observations suggest an active function for the immune system to control tumor growth, thus laying the foundation for modern cancer immunotherapy. Today, harnessing the immune system to control cancer is proven effective and garnering momentum. Currently, immunotherapy is largely classified into two functional treatment groups: (1) those that amplify/reactivate host existing innate and adaptive tumor immunity including check point inhibitors, dendritic cell (DC) vaccines and cytokines; (2) those that involve the adoptive transfer of genetically manipulated immune cells to target tumor cells *in vivo* such as chimeric antigen receptor (CAR) T cells, genetically enhanced effector T cells and natural killer (NK) cells. We will focus primarily on T- and NK-cell adoptive immunotherapy in this perspective.

## Adoptive T-Cell Therapy (ACT)

Adoptive T-cell therapy represents an attractive viable option for the control of solid tumor growth for the following reasons: T cells can systemically home to tumor sites through the entire body and can cross the blood–brain barrier (BBB). By contrast, antibodies such as checkpoint inhibitors cannot effectively cross the BBB and are not consistently or adequately distributed deep inside solid tumors. To date, ACT with CAR T cells is the prevalent type of immunotherapy to treat solid tumors.

### Neuroblastoma (NB)

Neuroblastoma is the most common extracranial solid tumor of childhood and the third most common cause of pediatric cancer death (29–32). Despite conventional multimodal therapy, patients with high-risk NB have a poor prognosis due to high relapse rate (33). Since 1999, tremendous efforts and funds have been dedicated to discovering and testing various forms of immunotherapy to control refractory/recurrent NB (Table 1). To date the most studied NB-associated antigen (NBAA) identified is the ganglioside GD2 expressed on a subset of NB tumor cells (34). This discovery heralds in the era of GD2-based immunotherapy to treat human NB. The current standard of care for refractory/recurrent NB in human is anti-GD2 therapy, which recognizes and binds GD2 (34–39). However, because GD2 is also expressed on pain fibers, this therapy has side effects that include severe pain requiring continuous opiate infusions (34). Because of the paucity of identified NBAAs, all CAR clinical trials are GD2-based or variations thereof (38, 40–45) (Table 1). It is still early to know whether these GD2-dependent CAR trials will yield objective clinical responses.

**Table 1:** Present and past adoptive immunotherapy trials for refractory/recurrent/relapsed neuroblastoma.

Approach	Status	NCT#	Sponsor	Year
Adoptive therapy of donor lymphocytes	Completed/no results	00003887	Fred Hutchinson Cancer Research Center	1999–2011
Adoptive therapy of autologous lymphocytes	Completed/no results	00006480	Fred Hutchinson Cancer Research Center	2000–2010
Adoptive therapy of autologous lymphocytes with EBV-lymphoblastoid vaccine	Unknown/no results	00101309	Milton S. Hershey Medical Center	2005–2007
Adoptive therapy of donor-derived tri-virus specific cytotoxic T cells	Completed/no results	01460901	Children’s Mercy Hospital Kansas City	2011–2015
Adoptive therapy of activated autologous chimeric GD2-iCas9 CAR T cells	Ongoing/no results	01822652	Baylor College of Medicine	2013–present
Adoptive therapy of CAR T cells expressing anti-GD2	Completed/no results	02107963	National Cancer Institute	2014–2017
Adoptive therapy of CAR T cells expressing anti-CD171	Recruiting	02311621	Seattle children’s Hospital	2014–present
Adoptive therapy of activated bispecific GD2 CAR T cells	Recruiting	02173093	Barbara Ann Karmanos Cancer Institute	2014–present
Infusion of haploidentical NK cells	Terminated/no results	00698009	MD Anderson Cancer Center	2008–2012
Infusion of allogeneic NK cells, humanized anti-GD2, and standard chemotherapy	Ongoing/no results	00877110	Memorial Sloan Kettering Cancer Center	2009–present
Infusion of allogeneic NK cells, humanized anti-GD2, and standard chemotherapy	Ongoing/no results	01576692	St. Jude Children’s Research Hospital	2012–present
Infusion of *in vitro*-activated/expanded NK cells	Completed/no results	01875601	National Cancer Institute (NCI)	2013–2016
Infusion of allogeneic NK cells from an acceptable parent	Recruiting	01857934	St. Jude Children’s Research Hospital	2013–present
Infusion of donor NK cells following haploidentical hematopoietic cell transplant	Recruiting	02100891	Monica Thakar Medical College of Wisconsin	2014–present
Infusion of CD133^+^ autologous stem cells followed by haploidentical NK cells	Recruiting	02130869	St. Jude Children’s Research Hospital	2014–present
Infusion of autologous expanded NK cells and anti-GD2	Not yet recruiting	02573896	New Approaches to Neuroblastoma Therapy Consortium	2015–present
Infusion of allogeneic NK cells and humanized anti-GD2	Recruiting	02650648	Memorial Sloan Kettering Cancer Center	2016–present
Infusion of *in vitro*-activated/expanded haploidentical NK cells and anti-GD2-IL2	Not yet recruiting	03209869	University of Wisconsin, Madison	2017

*EBV, Epstein–Barr virus; CAR, chimeric antigen receptor; GD2, glanglioside GD2; NK, natural killer; iCas9, inducible caspase 9*.

The antigen 4Ig-B7-H3 (CD276), a member of the B7 family of immune regulators such as CD80, CD86, PD-L1, PD-L2 and ICOSL, is expressed in a subset of NB tumors (46–49). In mouse and human, 4Ig-B7-H3 is expressed on many normal tissues such as spleen, lymph nodes, thymus and fetal liver as well as other tumors. However, its expression is induced on DCs and macrophages by inflammatory cytokines. 4Ig-B7-H3 binds to a yet to be identified cognate receptor induced on activated T and NK cells, and blockade of this interaction results in reduced interferon γ production and loss of cytotoxic activity of these cells. In mice, deficiency or blockade of B7-H3 leads to improved antitumor immunity suggesting that B7-H3 checkpoint may serve as a novel target for immunotherapy against cancers (50). Indeed, a mouse mAb anti-B7-H3 conjugated to iodine 131 (131I-burtomab) has been designed to bind and directly kill NB cells. 131I-burtomab is recently designated a breakthrough drug to treat metastatic NB by the Food and Drug Administration and a clinical trial has been filed (NCT03275402). The immune status of NB tumors treated with 131I-burtomab has not been published. It would be interesting to know whether the antitumor activity of burtomab results from direct killing of tumor cells or activated DCs thus reactivating existing anti-NB immunity. Although no published data are available to show the feasibility of B7-H3 CAR T cells for the treatment of human NB, two patents have been filed for such an invention (US Application No. 14/779,586; US Application No. 61/805,001; PCT Application No. PCT/US2014/031543 and PCT Application No. PCT/US2016/050887; US Application No. 62/216,447). It remains to be determined whether B7-H3 CAR T cells can inhibit NB tumor growth and whether the potential antitumor activity of B7-H3 CAR T cells is due to killing of intratumoral suppressive DCs/macrophages or tumor cells or both.

The anaplastic lymphoma kinase (ALK or CD246) is a receptor protein tyrosine kinase predominantly expressed in the central nervous system (CNS) and peripheral nervous system in mouse and human suggesting its role in normal brain development and function (51). A series of studies show that *Alk* is frequently mutated (mainly ALK^R1275Q^ and ALK^F1174L^) and duplicated in high-risk NB tumors (52–58). The ALK^R1275Q^ mutation results in a constitutively active kinase suggesting a role for ALK in NB development. However, mice harboring human ALK^R1275Q^ or ALK^F1174L^ alone do not develop aggressive NB irrespective of genetic background (53, 57). In the contrary, animals having both MYCN amplification and ALK^R1275Q^ or ALK^F1174L^ mutation succumb to NB at a higher rate (53, 57). These findings suggest that mutations in *Alk* are necessary but not sufficient to drive aggressive NB development. Because ALK is a cell surface kinase, developing CAR T cells targeting ALK has been suggested. Indeed, in a xenogeneic NSG mouse model for NB, human ALK CAR T cells can eradicate ALK-positive tumors; both tumor antigen and receptor density governs the efficacy of these CAR T cells (59). Clinical trials have not been initiated.

Although CAR T-cell therapy is being propelled to the forefront, problems exist that need further investigation. Production of CAR T cells requires the identification of tumor-associated antigen (TAA), generation of an antibody or T-cell receptor (TCR) capable of recognizing the TAA, cloning of genes encoding the antibody or TCR to be introduced into isolated tumor-infiltrating lymphocytes or haploidentical T cells. For most pediatric solid tumors, the identity of TAAs is still unknown and neoantigen load is low thus limiting the use of CAR T cells for this group. In patients with solid tumors for which TAAs have been identified, the use of CAR T cells has proven less effective than in patients with fluid tumors. Recent data are showing a previously unpredicted phenomenon observed in patients treated with CAR T cells: the emergence of tumor cells that have lost expression of the TAA targeted by CAR T cells, undoubtedly due to negative selection imposed by CAR therapy (60–62). New evidence demonstrates that CAR T cells once in the tumor microenvironment (TME) may suffer from exhaustion caused by suppressor cells including myeloid-derived suppressor cells or CD4^+^ regulatory T (Treg) cells present in the TME (63–65). Perhaps a more dangerous issue arisen is the development of cytokine release syndrome (CRS) (66, 67) and neurologic toxicity observed in patients undergoing CAR therapy (68).

To circumvent problems posed by CAR T cells, we propose that perhaps the most effective strategy to control solid tumor growth is one that does not require identifying TAAs and corresponding tumor-reactive CD8^+^ T cells, can enhance effector activity of CD8^+^ T cells, and can simultaneously eliminate immune suppression within the TME. Our recent studies suggest that such an approach is attainable. We show that by targeting the histone reader *Cbx3*/HP1γ, we can enhance the tumor killing capacity of effector CD8^+^ T cells (69, 70). As a result, adoptive transfer of *Cbx3*/HP1γ-deficient CD8^+^ effector T cells alone into wild type (wt) tumor-bearing mice greatly reduces NB growth. Within the NB TME of *Cbx3*/HP1γ-deficient mice or wt mice treated with *Cbx3*/HP1γ-deficient CD8^+^ T cells, we detect an increase of *Klrk1*/NKG2D^+^ infiltrating CD8^+^ effector T cells and a decrease in CD4^+^ Treg cells. PD-1 and *Pdl1* expression is not altered in CD8^+^ T cells or NB tumors, respectively. Moreover, *Cbx3*/HP1γ-deficient CD8^+^ T cells appear to have overcome exhaustion. These findings suggest that targeting *Cbx3*/HP1γ can represent an alternative and rational therapeutic approach to control NB as well as other solid tumors.

### Other Pediatric Solid Tumors (CNS Tumors, Sarcomas, and Nasopharyngeal Sarcomas)

As for NB, there is a dearth of identified TAAs available for the formulation of CAR therapy to treat most pediatric solid tumors (71–73). Tumor immunity against pediatric solid tumors is not completely understood. The human epidermal growth factor receptor 2 (HER2) is expressed on pediatric as well as adult glioblastoma, glioma, and medulloblastoma tumors, and overexpression of HER2 has been associated with poorer prognosis. In animal models, HER2 CAR T cells efficiently cause the regression of CNS tumors. These preclinical studies have paved the way for a few HER2-based CAR clinical trials (74–77) (Table 2); results of these trials are not yet available. It would be crucial to determine whether HER2-targeted CAR therapy will induce the emergence of HER2-negative tumors as has been shown in animal models and in patients receiving CD19 targeted CAR therapy (60, 61) or will cause CRS as in adults (67).

**Table 2:** Present and past adoptive immunotherapy for pediatric central nervous system tumors, sarcomas, and nasopharyngeal carcinomas.

Approach	Status	NCT#	Sponsor	Year
Adoptive therapy with autologous T cells following tumor vaccine	Completed (Ref)	00001566	National Cancer Institute (NCI)	1999–2012
Adoptive therapy with autologous T cells followed by DC vaccine	Completed/no results	00001564	National Cancer Institute (NCI)	1999–2014
Adoptive therapy with allogeneic EBV-specific T cells	Recruiting	00002663	Atara Biotherapeutics	1999–present
Adoptive therapy with autologous EBV-specific CTLs	Completed/no results	00516087	Baylor College of Medicine	2007–2017
Adoptive therapy with EBV-specific CTLs expressing HER2 CAR	Ongoing	00889954	Baylor College of Medicine	2009–present
Adoptive therapy with HER2/CD28 CAR T cells	Recruiting	00902044	Baylor College of Medicine	2009–present
Adoptive therapy with autologous EBV-specific CTLs	Completed/no results	00953420	Baylor College of Medicine	2009–2017
Adoptive therapy with CMV-specific CTLs expressing HER2 CAR	Ongoing	01109095	Baylor College of Medicine	2010–present
Adoptive therapy with haploidentical EBV-specific CTLs	Ongoing	01447056	Baylor College of Medicine	2011–present
Adoptive therapy with CD22 CAR T cells	Recruiting	02315612	National Cancer Institute (NCI)	2014–present
Adoptive therapy with glycan 3-specific autologous CAR T cells	Not yet recruiting	02932956	Baylor College of Medicine	2016–present
Adoptive therapy with NY-ESO-1 TCR transduced PBMC and NY-ESO-1 DC vaccine	Recruiting	02775292	Jonsson Comprehensive Cancer Center	2016–present
Infusion of autologous NK cells following peripheral blood stem cell transplant	Ongoing/no results	01287104	National Cancer Institute (NCI)	2011–present
Infusion of *in vitro*-activated/expanded NK cells	Completed/no results	01875601	National Cancer Institute (NCI)	2013–2016
Infusion of *in vitro*-activated/expanded NK cells	Completed/no results	01875601	National Cancer Institute (NCI)	2013–2016
Infusion of CD133+ autologous stem cells followed by haploidentical NK cells	Recruiting	02130869	St. Jude Children’s Research Hospital	2014–present
Infusion of donor NK cells following haploidentical hematopoietic cell transplant	Recruiting	02100891	Monica Thakar Medical College of Wisconsin	2014–2016

*EBV, Epstein–Barr virus; HER2, human epidermal growth factor receptor 2; CMV, cytomegalovirus*.

Pediatric nasopharyngeal carcinoma patients with local-regional bulky and metastatic disease have a poor prognosis (78). It is a rare tumor that is almost always associated with Epstein–Barr virus (EBV) (78, 79), and EBV-specific cytotoxic T lymphocytes (CTLs) can be found in individuals infected with this ubiquitous virus. These findings lead to the design of EBV-based T-cell therapy. In adults, ACT with EBV-specific CTLs is more effective in patients with low disease burden while results for pediatric trials are not available (80–83) (Table 2).

Prognosis for pediatric patients with recurrent/refractory sarcomas is poor, the survival rate ranges from 10 to 30% (73). For this group of children, few immunotherapy clinical trials are being tested, and past trials using autologous T cells (NCT00001566 and NCT 00001564) have not yielded much information to advance the field (Table 2).

Some studies have shown that the cancer-testes antigen NY-ESO-1 is expressed on a subset of pediatric tumors, which lead to a trial using T cells engineered to express NY-ESO-1-specific TCR (NCT02775292) (84) (Table 2). HER2 CAR therapy has also been proposed to treat sarcomas (85). CAR T cells targeting glypican-3, a proteoglycan expressed on a small number of solid tumors (86), are being tested in clinical trials to treat pediatric solid tumors (87) (NCT02932956). Data are not yet available for these trials.

The lack of available immunotherapy for pediatric solid tumors may be due to the paucity of identified TAAs, and few basic studies designed to understand tumor immunity during development from infancy to young adulthood in either mouse or human. As a result, most of these trials are based on those that have been designed to treat adult solid tumors.

## Adoptive NK- and Natural Killer T (NKT)-Cell Therapy

Natural killer cells and NKT cells have been shown to play crucial roles in antitumor immunity by directly killing tumor cells or indirectly through antibody-dependent cellular cytotoxicity. Based on results from preclinical studies (88–90), several clinical trials have been initiated to test the ability of *in vitro*-activated/expanded or engineered NK cells and NKT cells to control pediatric solid tumors including NB (91, 92) (Tables 1 and 2). Results of these trials are not yet available to indicate whether NK- or NKT-cell therapy would be a viable option.

Nonetheless, clinical data have demonstrated that despite the large number of NK cells infused, the antitumor effects of these cells have been modest in adults. NK and NKT cells express a number of inhibitory receptors that bind to MHC class I and other molecules. Additionally, NK and NKT cells are sensitive to various inhibitory molecules within the TME (93). Moreover, we show that the frequency of NK and NKT cells in NB tumors is low, and no differences are detected in tumors from wt or *Cbx3*/HP1γ-deficient mice yet NB tumor growth is greatly abrogated in *Cbx3*/HP1γ-deficient mice (70). Our findings imply that NK or NKT cells may not play an important role in controlling NB tumor growth.

## Discussion and Future Directions

In the past 40–50 years, pediatric oncologists have made significant, basic advances toward our understanding of molecular pathways driving the development of tumors in children. This achievement hinges on the belief that cancer pediatric patients represent a distinct demographic group, and the biology of their tumors is fundamentally different from that of adults.

Adult cancer immunotherapy is experiencing a renaissance while that of children is still at its infancy. The momentum that drives adult cancer immunotherapy is built upon decades of basic research designed to understand how an adult immune system responses to tumors developing within an adult host. Thus, there is a need to recognize that pediatric cancer patients represent a distinct demographic group whose immune system is fundamentally different than that of mature adults.

Fetal and adult T cells are distinct populations that arise from different hematopoietic stem cell populations present at different developmental stages (94), and human NK cells follow similar developmental evolution (95, 96). Notably, fetal CD4^+^ T cells are poised to differentiate into CD4^+^ Treg cells upon allogeneic stimulation. Indeed, human fetus and cord blood (CB) contains an abundance of phenotypically naïve CD25^+^CD4^+^ Treg cells, but functionally mature, capable of suppressing T- and NK-cell proliferation and function (97–101). Similar suppressive mechanism is observed in the mouse fetus (102). Thus, T- and NK-cell lineages in the developing human or mouse are biased toward immune tolerance mediated by active suppression of early immunity; in some instances, this suppression persists at least until early adulthood (101). The tolerogenic tendency of fetal/neonatal immune system can be attributed to marked differences in response to alloantigens between human fetal and adult DCs. Fetal DCs strongly promote Treg-cell induction and inhibit T-cell tumor-necrosis factor-α production when cultured with alloantigens (103). This may explain why CAR T cells once in the NB TME often suffer from exhaustion. In addition to functional disparities, neonatal and adult immune systems differ quantitatively. Overall, there is a greater number of circulating CD4^+^ T cells and a lower number of CD8^+^ T cells in neonates compared to adults (104). Consequently, the ratio of CD4:CD8 is higher in neonates than adults. Together these results suggest that for some children, the persistence of fetal immune suppression, mediated by fetal CD4^+^ Treg cells, and the lower number of CD8^+^ T cells may render their immune system incapable of surveilling and eradicating tumor. Therefore in the future, it might be essential to study the effects of persistent fetal immune suppression on tumor development and growth. If the persistence of fetal immune suppression does influence antitumor immunity in pediatric patients, it would be crucial to determine the developmental age at which intervention can be mounted to prevent tumorigenesis and growth without breaking tolerance. Clinically, it might be important to collect data on the immune status of children bearing solid tumors in addition to dissecting their tumor immune environment. Results from these studies might help direct the design of T- and NK-cell therapies that can circumvent suppression and prevent exhaustion induction.

In adult tumors, mutation load appears to correlate with tumor immunity. However, the number of somatic mutations in pediatric solid tumors is low. In the future, it would be necessary to determine mechanisms controlling tumor immunity independent of somatic mutation loads in pediatric patients.

For this demographic group, perhaps the most effective strategy to control solid tumor growth is one that does not require identifying TAAs or neoantigens and their corresponding reactive CD8^+^ T cells, can enhance effector activity of CD8^+^ T cells, and can simultaneously eliminate immune suppression within the TME.

We believe these are crucial issues that need to be addressed in order to move the field of pediatric adoptive cancer immunotherapy to the fore. Until there is a will to allow for the funding of such studies and those that are outside traditional belief, children with cancers will continue to be treated as adults, and may not benefit from the cancer immunotherapy renaissance.

## Author Contributions

TPL and THT conceived and cowrote this manuscript.

## Conflict of Interest Statement

The authors declare that the research was conducted in the absence of any commercial or financial relationships that could be construed as a potential conflict of interest.

## References

[1] Delattre O Zucman J Plougastel B Desmaze C Melot T Peter M Gene fusion with an ETS DNA-binding domain caused by chromosome translocation in human tumours. Nature (1992) 359(6391):162–5. 10.1038/359162a0 1522903

[2] Grady-Leopardi EF Schwab M Ablin AR Rosenau W. Detection of N-myc oncogene expression in human neuroblastoma by in situ hybridization and blot analysis: relationship to clinical outcome. Cancer Res (1986) 46(6):3196–9. 2421891

[3] Jha P Suri V Singh G Jha P Purkait S Pathak P Characterization of molecular genetic alterations in GBMs highlights a distinctive molecular profile in young adults. Diagn Mol Pathol (2011) 20(4):225–32. 10.1097/PDM.0b013e31821c30bc 22089350

[4] Parsons DW Li M Zhang X Jones S Leary RJ Lin JC The genetic landscape of the childhood cancer medulloblastoma. Science (2011) 331(6016):435–9. 10.1126/science.1198056 21163964 PMC3110744

[5] Roberts KG Li Y Payne-Turner D Harvey RC Yang YL Pei D Targetable kinase-activating lesions in Ph-like acute lymphoblastic leukemia. N Engl J Med (2014) 371(11):1005–15. 10.1056/NEJMoa1403088 25207766 PMC4191900

[6] Roberts KG Morin RD Zhang J Hirst M Zhao Y Su X Genetic alterations activating kinase and cytokine receptor signaling in high-risk acute lymphoblastic leukemia. Cancer Cell (2012) 22(2):153–66. 10.1016/j.ccr.2012.06.005 22897847 PMC3422513

[7] Schwab M Alitalo K Klempnauer KH Varmus HE Bishop JM Gilbert F Amplified DNA with limited homology to myc cellular oncogene is shared by human neuroblastoma cell lines and a neuroblastoma tumour. Nature (1983) 305(5931):245–8. 10.1038/305245a0 6888561

[8] Galili N Davis RJ Fredericks WJ Mukhopadhyay S Rauscher FJ III Emanuel BS Fusion of a fork head domain gene to PAX3 in the solid tumour alveolar rhabdomyosarcoma. Nat Genet (1993) 5(3):230–5. 10.1038/ng1193-230 8275086

[9] Smith MA Seibel NL Altekruse SF Ries LA Melbert DL O’Leary M Outcomes for children and adolescents with cancer: challenges for the twenty-first century. J Clin Oncol (2010) 28(15):2625–34. 10.1200/JCO.2009.27.0421 20404250 PMC2881732

[10] Cohen KJ Pollack IF Zhou T Buxton A Holmes EJ Burger PC Temozolomide in the treatment of high-grade gliomas in children: a report from the Children’s Oncology Group. Neuro Oncol (2011) 13(3):317–23. 10.1093/neuonc/noq191 21339192 PMC3064602

[11] MacDonald TJ Aguilera D Kramm CM. Treatment of high-grade glioma in children and adolescents. Neuro Oncol (2011) 13(10):1049–58. 10.1093/neuonc/nor092 21784756 PMC3177659

[12] Feun LG Savaraj N Landy HJ. Drug resistance in brain tumors. J Neurooncol (1994) 20(2):165–76. 10.1007/bf01052726 7807193

[13] Phillips PC Antineoplastic drug resistance in brain tumors. Neurol Clin (1991) 9(2):383–404. 1682794

[14] McNeil DE Cote TR Clegg L Rorke LB. Incidence and trends in pediatric malignancies medulloblastoma/primitive neuroectodermal tumor: a SEER update. Surveillance Epidemiology and End Results. Med Pediatr Oncol (2002) 39(3):190–4. 10.1002/mpo.10121 12210449

[15] Moreno L Marshall LV Pearson AD. At the frontier of progress for paediatric oncology: the neuroblastoma paradigm. Br Med Bull (2013) 108:173–88. 10.1093/bmb/ldt033 24211816

[16] Lagmay JP Krailo MD Dang H Kim A Hawkins DS Beaty O III Outcome of patients with recurrent osteosarcoma enrolled in seven phase II trials through children’s cancer group, pediatric oncology group, and children’s oncology group: learning from the past to move forward. J Clin Oncol (2016) 34(25):3031–8. 10.1200/JCO.2015.65.5381 27400942 PMC5012712

[17] Shankar AG Ashley S Craft AW Pinkerton CR Outcome after relapse in an unselected cohort of children and adolescents with Ewing sarcoma. Med Pediatr Oncol (2003) 40(3):141–7. 10.1002/mpo.10248 12518341

[18] Taylor BS Barretina J Maki RG Antonescu CR Singer S Ladanyi M. Advances in sarcoma genomics and new therapeutic targets. Nat Rev Cancer (2011) 11(8):541–57. 10.1038/nrc3087 21753790 PMC3361898

[19] Duffner PK Armstrong FD Chen L Helton KJ Brecher ML Bell B Neurocognitive and neuroradiologic central nervous system late effects in children treated on Pediatric Oncology Group (POG) P9605 (standard risk) and P9201 (lesser risk) acute lymphoblastic leukemia protocols (ACCL0131): a methotrexate consequence? A report from the Children’s Oncology Group. J Pediatr Hematol Oncol (2014) 36(1):8–15. 10.1097/MPH.0000000000000000 24345882 PMC4465396

[20] Galloway TJ Indelicato DJ Amdur RJ Swanson EL Smith AA Marcus RB Jr Second tumors in pediatric patients treated with radiotherapy to the central nervous system. Am J Clin Oncol (2012) 35(3):279–83. 10.1097/COC.0b013e318210f533 21383606

[21] Applebaum MA Vaksman Z Lee SM Hungate EA Henderson TO London WB Neuroblastoma survivors are at increased risk for second malignancies: a report from the International Neuroblastoma Risk Group Project. Eur J Cancer (2017) 72:177–85. 10.1016/j.ejca.2016.11.022 28033528 PMC5258837

[22] Lancashire ER Frobisher C Reulen RC Winter DL Glaser A Hawkins MM. Educational attainment among adult survivors of childhood cancer in Great Britain: a population-based cohort study. J Natl Cancer Inst (2010) 102(4):254–70. 10.1093/jnci/djp498 20107164

[23] Frobisher C Gurung PM Leiper A Reulen RC Winter DL Taylor AJ Risk of bladder tumours after childhood cancer: the British Childhood Cancer Survivor Study. BJU Int (2010) 106(7):1060–9. 10.1111/j.1464-410X.2010.09224.x 20184574

[24] Reulen RC Winter DL Frobisher C Lancashire ER Stiller CA Jenney ME Long-term cause-specific mortality among survivors of childhood cancer. JAMA (2010) 304(2):172–9. 10.1001/jama.2010.923 20628130

[25] Taylor AJ Little MP Winter DL Sugden E Ellison DW Stiller CA Population-based risks of CNS tumors in survivors of childhood cancer: the British Childhood Cancer Survivor Study. J Clin Oncol (2010) 28(36):5287–93. 10.1200/JCO.2009.27.0090 21079138 PMC4809645

[26] Kuhlthau KA Pulsifer MB Yeap BY Rivera Morales D Delahaye J Hill KS Prospective study of health-related quality of life for children with brain tumors treated with proton radiotherapy. J Clin Oncol (2012) 30(17):2079–86. 10.1200/JCO.2011.37.0577 22565004 PMC3397695

[27] Coley WB The treatment of malignant tumors by repeated inoculations of erysipelas. With a report of ten original cases. 1893. Clin Orthop Relat Res (1991) 262:3–11. 1984929

[28] Coley WB The treatment of inoperable sarcoma by bacterial toxins (the mixed toxins of the *Streptococcus erysipelas* and the *Bacillus prodigiosus*). Proc R Soc Med (1910) 3(Surg Sect):1–48. 10.1177/003591571000301601 PMC1961042 19974799

[29] Seeger RC. Immunology and immunotherapy of neuroblastoma. Semin Cancer Biol (2011) 21(4):229–37. 10.1016/j.semcancer.2011.09.012 21971567 PMC3658311

[30] Speleman F Park JR Henderson TO. Neuroblastoma: a tough nut to crack. Am Soc Clin Oncol Educ Book (2016) 35:e548–57. 10.14694/EDBK_159169 27249766

[31] Cheung NK Dyer MA. Neuroblastoma: developmental biology, cancer genomics and immunotherapy. Nat Rev Cancer (2013) 13(6):397–411. 10.1038/nrc3526 23702928 PMC4386662

[32] Sharp SE Gelfand MJ Shulkin BL. Pediatrics: diagnosis of neuroblastoma. Semin Nucl Med (2011) 41(5):345–53. 10.1053/j.semnuclmed.2011.05.001 21803184

[33] Li R Polishchuk A DuBois S Hawkins R Lee SW Bagatell R Patterns of relapse in high-risk neuroblastoma patients treated with and without total body irradiation. Int J Radiat Oncol Biol Phys (2017) 97(2):270–7. 10.1016/j.ijrobp.2016.10.047 28068235

[34] Suzuki M Cheung NK. Disialoganglioside GD2 as a therapeutic target for human diseases. Expert Opin Ther Targets (2015) 19(3):349–62. 10.1517/14728222.2014.986459 25604432

[35] Yang RK Sondel PM Anti-GD2 strategy in the treatment of neuroblastoma. Drugs Future (2010) 35(8):665 10.1358/dof.2010.035.08.1513490 21037966 PMC2964668

[36] Yu AL Gilman AL Ozkaynak MF London WB Kreissman SG Chen HX Anti-GD2 antibody with GM-CSF, interleukin-2, and isotretinoin for neuroblastoma. N Engl J Med (2010) 363(14):1324–34. 10.1056/NEJMoa0911123 20879881 PMC3086629

[37] Cheung NK Cheung IY Kushner BH Ostrovnaya I Chamberlain E Kramer K Murine anti-GD2 monoclonal antibody 3F8 combined with granulocyte-macrophage colony-stimulating factor and 13-cis-retinoic acid in high-risk patients with stage 4 neuroblastoma in first remission. J Clin Oncol (2012) 30(26):3264–70. 10.1200/JCO.2011.41.3807 22869886 PMC3434986

[38] Hoseini SS Dobrenkov K Pankov D Xu XL Cheung N-KV Bispecific antibody does not induce T-cell death mediated by chimeric antigen receptor against disialoganglioside GD2. Oncoimmunology (2017) 6(6):e1320625 10.1080/2162402X.2017.1320625 28680755 PMC5486173

[39] Navid F Sondel PM Barfield R Shulkin BL Kaufman RA Allay JA Phase I trial of a novel anti-GD2 monoclonal antibody, Hu14.18K322A, designed to decrease toxicity in children with refractory or recurrent neuroblastoma. J Clin Oncol (2014) 32(14):1445–52. 10.1200/JCO.2013.50.4423 24711551 PMC4017710

[40] Xu H Cheng M Guo H Chen Y Huse M Cheung NK. Retargeting T cells to GD2 pentasaccharide on human tumors using bispecific humanized antibody. Cancer Immunol Res (2015) 3(3):266–77. 10.1158/2326-6066.CIR-14-0230-T 25542634 PMC4351131

[41] Gargett T Yu W Dotti G Yvon ES Christo SN Hayball JD GD2-specific CAR T cells undergo potent activation and deletion following antigen encounter but can be protected from activation-induced cell death by PD-1 blockade. Mol Ther (2016) 24(6):1135–49. 10.1038/mt.2016.63 27019998 PMC4923328

[42] Heczey A Louis CU Savoldo B Dakhova O Durett A Grilley B CAR T cells administered in combination with lymphodepletion and PD-1 inhibition to patients with neuroblastoma. Mol Ther (2017) 25(9):2214–24. 10.1016/j.ymthe.2017.05.012 28602436 PMC5589058

[43] Prapa M Caldrer S Spano C Bestagno M Golinelli G Grisendi G A novel anti-GD2/4-1BB chimeric antigen receptor triggers neuroblastoma cell killing. Oncotarget (2015) 6(28):24884–94. 10.18632/oncotarget.4670 26298772 PMC4694800

[44] Nishio N Diaconu I Liu H Cerullo V Caruana I Hoyos V Armed oncolytic virus enhances immune functions of chimeric antigen receptor-modified T cells in solid tumors. Cancer Res (2014) 74(18):5195–205. 10.1158/0008-5472.CAN-14-0697 25060519 PMC4167556

[45] Pule MA Savoldo B Myers GD Rossig C Russell HV Dotti G Virus-specific T cells engineered to coexpress tumor-specific receptors: persistence and antitumor activity in individuals with neuroblastoma. Nat Med (2008) 14(11):1264–70. 10.1038/nm.1882 18978797 PMC2749734

[46] Castriconi R Dondero A Augugliaro R Cantoni C Carnemolla B Sementa AR Identification of 4Ig-B7-H3 as a neuroblastoma-associated molecule that exerts a protective role from an NK cell-mediated lysis. Proc Natl Acad Sci U S A (2004) 101(34):12640–5. 10.1073/pnas.0405025101 15314238 PMC515110

[47] Steinberger P Majdic O Derdak SV Pfistershammer K Kirchberger S Klauser C Molecular characterization of human 4Ig-B7-H3, a member of the B7 family with four Ig-like domains. J Immunol (2004) 172(4):2352–9. 10.4049/jimmunol.172.4.2352 14764704

[48] Sun M Richards S Prasad DVR Mai XM Rudensky A Dong C. Characterization of mouse and human B7-H3 genes. J Immunol (2002) 168(12):6294–7. 10.4049/jimmunol.168.12.6294 12055244

[49] Chapoval AI Ni J Lau JS Wilcox RA Flies DB Liu D B7-H3: a costimulatory molecule for T cell activation and IFN-[gamma] production. Nat Immunol (2001) 2(3):269–74. 10.1038/85339 11224528

[50] Lee Y-H Martin-Orozco N Zheng P Li J Zhang P Tan H Inhibition of the B7-H3 immune checkpoint limits tumor growth by enhancing cytotoxic lymphocyte function. Cell Res (2017) 27(8):1034–45. 10.1038/cr.2017.90 28685773 PMC5539354

[51] Morris S Kirstein M Valentine M Dittmer K Shapiro D Saltman D Fusion of a kinase gene, ALK, to a nucleolar protein gene, NPM, in non-Hodgkin’s lymphoma. Science (1994) 263(5151):1281–4. 10.1126/science.8122112 8122112

[52] Chen Y Takita J Choi YL Kato M Ohira M Sanada M Oncogenic mutations of ALK kinase in neuroblastoma. Nature (2008) 455(7215):971–4. 10.1038/nature07399 18923524

[53] Berry T Luther W Bhatnagar N Jamin Y Poon E Sanda T The ALK(F1174L) mutation potentiates the oncogenic activity of MYCN in neuroblastoma. Cancer Cell (2012) 22(1):117–30. 10.1016/j.ccr.2012.06.001 22789543 PMC3417812

[54] George RE Sanda T Hanna M Frohling S Ii WL Zhang J Activating mutations in ALK provide a therapeutic target in neuroblastoma. Nature (2008) 455(7215):975–8. 10.1038/nature07397 18923525 PMC2587486

[55] Mosse YP Laudenslager M Longo L Cole KA Wood A Attiyeh EF Identification of ALK as a major familial neuroblastoma predisposition gene. Nature (2008) 455(7215):930–5. 10.1038/nature07261 18724359 PMC2672043

[56] Pugh TJ Morozova O Attiyeh EF Asgharzadeh S Wei JS Auclair D The genetic landscape of high-risk neuroblastoma. Nat Genet (2013) 45(3):279–84. 10.1038/ng.2529 23334666 PMC3682833

[57] Ueda T Nakata Y Yamasaki N Oda H Sentani K Kanai A ALKR1275Q perturbs extracellular matrix, enhances cell invasion and leads to the development of neuroblastoma in cooperation with MYCN. Oncogene (2016) 35(34):4447–58. 10.1038/onc.2015.519 26829053

[58] Janoueix-Lerosey I Lequin D Brugieres L Ribeiro A de Pontual L Combaret V Somatic and germline activating mutations of the ALK kinase receptor in neuroblastoma. Nature (2008) 455(7215):967–70. 10.1038/nature07398 18923523

[59] Walker AJ Majzner RG Zhang L Wanhainen K Long AH Nguyen SM Tumor antigen and receptor densities regulate efficacy of a chimeric antigen receptor targeting anaplastic lymphoma kinase. Mol Ther (2017) 25(9):2189–201. 10.1016/j.ymthe.2017.06.008 28676342 PMC5589087

[60] Haso W Lee DW Shah NN Stetler-Stevenson M Yuan CM Pastan IH Anti-CD22-chimeric antigen receptors targeting B-cell precursor acute lymphoblastic leukemia. Blood (2013) 121(7):1165–74. 10.1182/blood-2012-06-438002 23243285 PMC3575759

[61] Lee DW Kochenderfer JN Stetler-Stevenson M Cui YK Delbrook C Feldman SA T cells expressing CD19 chimeric antigen receptors for acute lymphoblastic leukaemia in children and young adults: a phase 1 dose-escalation trial. Lancet (2015) 385(9967):517–28. 10.1016/S0140-6736(14)61403-3 25319501 PMC7065359

[62] Hegde M Mukherjee M Grada Z Pignata A Landi D Navai SA Tandem CAR T cells targeting HER2 and IL13Ralpha2 mitigate tumor antigen escape. J Clin Invest (2016) 126(8):3036–52. 10.1172/JCI83416 27427982 PMC4966331

[63] Caruana I Savoldo B Hoyos V Weber G Liu H Kim ES Heparanase promotes tumor infiltration and antitumor activity of CAR-redirected T lymphocytes. Nat Med (2015) 21(5):524–9. 10.1038/nm.3833 25849134 PMC4425589

[64] Long AH Haso WM Shern JF Wanhainen KM Murgai M Ingaramo M 4-1BB costimulation ameliorates T cell exhaustion induced by tonic signaling of chimeric antigen receptors. Nat Med (2015) 21(6):581–90. 10.1038/nm.3838 25939063 PMC4458184

[65] Long AH Highfill SL Cui Y Smith JP Walker AJ Ramakrishna S Reduction of MDSCs with all-trans retinoic acid improves CAR therapy efficacy for sarcomas. Cancer Immunol Res (2016) 4(10):869–80. 10.1158/2326-6066.cir-15-0230 27549124 PMC5050151

[66] Lee DW Gardner R Porter DL Louis CU Ahmed N Jensen M Current concepts in the diagnosis and management of cytokine release syndrome. Blood (2014) 124(2):188–95. 10.1182/blood-2014-05-552729 24876563 PMC4093680

[67] Morgan RA Yang JC Kitano M Dudley ME Laurencot CM Rosenberg SA. Case report of a serious adverse event following the administration of T cells transduced with a chimeric antigen receptor recognizing ERBB2. Mol Ther (2010) 18(4):843–51. 10.1038/mt.2010.24 20179677 PMC2862534

[68] Neelapu SS Tummala S Kebriaei P Wierda W Gutierrez C Locke FL Chimeric antigen receptor T-cell therapy [mdash] assessment and management of toxicities. Nat Rev Clin Oncol (2017). 10.1038/nrclinonc.2017.148 PMC6733403 28925994

[69] Ha N Pham DH Shahsafaei A Naruse C Asano M Thai TH HP-1gamma controls high-affinity antibody response to T-dependent antigens. Front Immunol (2014) 5:271 10.3389/fimmu.2014.00271 24971082 PMC4053855

[70] Sun M Ha N Pham DH Frederick M Sharma B Naruse C Cbx3/HP1gamma deficiency confers enhanced tumor-killing capacity on CD8+ T cells. Sci Rep (2017) 7:42888 10.1038/srep42888 28220815 PMC5318867

[71] Bielamowicz K Khawja S Ahmed N Adoptive cell therapies for glioblastoma. Front Oncol (2013) 3:275 10.3389/fonc.2013.00275 24273748 PMC3823029

[72] Hegde M Bielamowicz KJ Ahmed N. Novel approaches and mechanisms of immunotherapy for glioblastoma. Discov Med (2014) 17(93):145–54. 24641957

[73] Hegde M Moll AJ Byrd TT Louis CU Ahmed N. Cellular immunotherapy for pediatric solid tumors. Cytotherapy (2015) 17(1):3–17. 10.1016/j.jcyt.2014.05.019 25082406 PMC4629832

[74] Ahmed N Brawley VS Hegde M Robertson C Ghazi A Gerken C Human epidermal growth factor receptor 2 (HER2)-specific chimeric antigen receptor-modified T cells for the immunotherapy of HER2-positive sarcoma. J Clin Oncol (2015) 33(15):1688–96. 10.1200/JCO.2014.58.0225 25800760 PMC4429176

[75] Ahmed N Ratnayake M Savoldo B Perlaky L Dotti G Wels WS Regression of experimental medulloblastoma following transfer of HER2-specific T cells. Cancer Res (2007) 67(12):5957–64. 10.1158/0008-5472.CAN-06-4309 17575166

[76] Ahmed N Salsman VS Kew Y Shaffer D Powell S Zhang YJ HER2-specific T cells target primary glioblastoma stem cells and induce regression of autologous experimental tumors. Clin Cancer Res (2010) 16(2):474–85. 10.1158/1078-0432.CCR-09-1322 20068073 PMC3682507

[77] Ahmed N Salsman VS Yvon E Louis CU Perlaky L Wels WS Immunotherapy for osteosarcoma: genetic modification of T cells overcomes low levels of tumor antigen expression. Mol Ther (2009) 17(10):1779–87. 10.1038/mt.2009.133 19532139 PMC2835000

[78] Chan AT Teo PM Johnson PJ. Nasopharyngeal carcinoma. Ann Oncol (2002) 13(7):1007–15. 10.1093/annonc/mdf179 12176778

[79] Raab-Traub N. Epstein-Barr virus in the pathogenesis of NPC. Semin Cancer Biol (2002) 12(6):431–41. 10.1016/S1044579X0200086X 12450729

[80] Comoli P Pedrazzoli P Maccario R Basso S Carminati O Labirio M Cell therapy of stage IV nasopharyngeal carcinoma with autologous Epstein-Barr virus-targeted cytotoxic T lymphocytes. J Clin Oncol (2005) 23(35):8942–9. 10.1200/JCO.2005.02.6195 16204009

[81] Straathof KC Leen AM Buza EL Taylor G Huls MH Heslop HE Characterization of latent membrane protein 2 specificity in CTL lines from patients with EBV-positive nasopharyngeal carcinoma and lymphoma. J Immunol (2005) 175(6):4137–47. 10.4049/jimmunol.175.6.4137 16148164

[82] Straathof KC Pule MA Yotnda P Dotti G Vanin EF Brenner MK An inducible caspase 9 safety switch for T-cell therapy. Blood (2005) 105(11):4247–54. 10.1182/blood-2004-11-4564 15728125 PMC1895037

[83] Louis CU Straathof K Bollard CM Gerken C Huls MH Gresik MV Enhancing the in vivo expansion of adoptively transferred EBV-specific CTL with lymphodepleting CD45 monoclonal antibodies in NPC patients. Blood (2009) 113(11):2442–50. 10.1182/blood-2008-05-157222 18971421 PMC2656271

[84] Jungbluth AA Antonescu CR Busam KJ Iversen K Kolb D Coplan K Monophasic and biphasic synovial sarcomas abundantly express cancer/testis antigen NY-ESO-1 but not MAGE-A1 or CT7. Int J Cancer (2001) 94(2):252–6. 10.1002/ijc.1451 11668506

[85] Rainusso N Brawley VS Ghazi A Hicks MJ Gottschalk S Rosen JM Immunotherapy targeting HER2 with genetically modified T cells eliminates tumor-initiating cells in osteosarcoma. Cancer Gene Ther (2012) 19(3):212–7. 10.1038/cgt.2011.83 22173710

[86] Kinoshita Y Tanaka S Souzaki R Miyoshi K Kohashi K Oda Y Glypican 3 expression in pediatric malignant solid tumors. Eur J Pediatr Surg (2015) 25(1):138–44. 10.1055/s-0034-1393961 25344940

[87] Bi Y Jiang H Wang P Song B Wang H Kong X Treatment of hepatocellular carcinoma with a GPC3-targeted bispecific T cell engager. Oncotarget (2017) 8(32):52866–76. 10.18632/oncotarget.17905 28881778 PMC5581077

[88] Seidel D Shibina A Siebert N Wels WS Reynolds CP Huebener N Disialoganglioside-specific human natural killer cells are effective against drug-resistant neuroblastoma. Cancer Immunol Immunother (2015) 64(5):621–34. 10.1007/s00262-015-1669-5 25711293 PMC11029162

[89] Esser R Muller T Stefes D Kloess S Seidel D Gillies SD NK cells engineered to express a GD2-specific antigen receptor display built-in ADCC-like activity against tumour cells of neuroectodermal origin. J Cell Mol Med (2012) 16(3):569–81. 10.1111/j.1582-4934.2011.01343.x 21595822 PMC3822932

[90] Liu Y Wu H-W Sheard MA Sposto R Somanchi SS Cooper LJN Growth and activation of natural killer cells ex vivo from children with neuroblastoma for adoptive cell therapy. Clin Cancer Res (2013) 19(8):2132–43. 10.1158/1078-0432.ccr-12-1243 23378384 PMC3658308

[91] Perez-Martinez A Leung W Munoz E Iyengar R Ramirez M Vicario JL KIR-HLA receptor-ligand mismatch associated with a graft-versus-tumor effect in haploidentical stem cell transplantation for pediatric metastatic solid tumors. Pediatr Blood Cancer (2009) 53(1):120–4. 10.1002/pbc.21955 19215002

[92] Song L Asgharzadeh S Salo J Engell K Wu HW Sposto R Valpha24-invariant NKT cells mediate antitumor activity via killing of tumor-associated macrophages. J Clin Invest (2009) 119(6):1524–36. 10.1172/JCI37869 19411762 PMC2689106

[93] Lanier LL. NK cell recognition. Annu Rev Immunol (2005) 23:225–74. 10.1146/annurev.immunol.23.021704.115526 15771571

[94] Mold JE Venkatasubrahmanyam S Burt TD Michaelsson J Rivera JM Galkina SA Fetal and adult hematopoietic stem cells give rise to distinct T cell lineages in humans. Science (2010) 330(6011):1695–9. 10.1126/science.1196509 21164017 PMC3276679

[95] Uksila J Lassila O Hirvonen T Toivanen P. Development of natural killer cell function in the human fetus. J Immunol (1983) 130(1):153–6. 6847879

[96] Schönberg K Fischer JC Kögler G Uhrberg M Neonatal NK-cell repertoires are functionally, but not structurally, biased toward recognition of self HLA class I. Blood (2011) 117(19):5152–6. 10.1182/blood-2011-02-334441 21415265

[97] Takahata Y Nomura A Takada H Ohga S Furuno K Hikino S CD25+CD4+ T cells in human cord blood: an immunoregulatory subset with naive phenotype and specific expression of forkhead box p3 (Foxp3) gene. Exp Hematol (2004) 32(7):622–9. 10.1016/j.exphem.2004.03.012 15246158

[98] Dalle J-H Menezes J Wagner E Blagdon M Champagne J Champagne MA Characterization of cord blood natural killer cells: implications for transplantation and neonatal infections. Pediatr Res (2005) 57(5 Pt 1):649–55. 10.1203/01.PDR.0000156501.55431.20 15718362

[99] Ivarsson MA Loh L Marquardt N Kekalainen E Berglin L Bjorkstrom NK Differentiation and functional regulation of human fetal NK cells. J Clin Invest (2013) 123(9):3889–901. 10.1172/JCI68989 23945237 PMC3754261

[100] Michaëlsson J Mold JE McCune JM Nixon DF Regulation of T cell responses in the developing human fetus. J Immunol (2006) 176(10):5741–8. 10.4049/jimmunol.176.10.5741 16670279

[101] Mold JE Michaëlsson J Burt TD Muench MO Beckerman KP Busch MP Maternal alloantigens promote the development of tolerogenic fetal regulatory T cells in utero. Science (2008) 322(5907):1562–5. 10.1126/science.1164511 19056990 PMC2648820

[102] Marcoe JP Lim JR Schaubert KL Fodil-Cornu N Matka M McCubbrey AL TGF-[beta] is responsible for NK cell immaturity during ontogeny and increased susceptibility to infection during mouse infancy. Nat Immunol (2012) 13(9):843–50. 10.1038/ni.2388 22863752 PMC3426626

[103] McGovern N Shin A Low G Low D Duan K Yao LJ Human fetal dendritic cells promote prenatal T-cell immune suppression through arginase-2. Nature (2017) 546(7660):662–6. 10.1038/nature22795 28614294 PMC6588541

[104] Maheshwari A Calhoun DA Developmental hematopoietic cell deficiencies in the neonatal immune system. In: Stiehm ER, editor. UpToDate. Waltham, MA (2017).

